# An Improved Mode of Managing Blisters

**Published:** 1845-02

**Authors:** John Key Robertson

**Affiliations:** Glasgow


*An Improved Mode of Managing Blisters. By John Key Robertson, M.D., Glasgow.*—The improvements I beg to suggest are the following. The common empl. canth. of the pharmacopœia is to be used:

I. It is generally spread on leather.

*It should never be spread on leather*, but on thin paper, (partially sized, or printing paper,) or upon soft linen. Leather, by the heat of many parts of the body, becomes dry, partially crisp, and with difficulty adheres to the skin, producing, even if the blister were otherwise good, an impossibility in many instances of acting well and generally over the whole part intended to be blistered, from not being over the whole extent of the blister in contact with the surface of the body. The linen or paper has no such objection, and clings easily and to anywhere. I invariably use *paper*, and straps to secure it, above these a small pad, and then a bandage.

II. It is generally spread very thick.

*It should be spread very thin*—a mere covering of the paper or linen is all that is requisite. The thickness partakes of the objection already mentioned with reference to leather; and as no more than the mere surface of the blister can act on the skin, to make a blister thick is, in addition to rendering it in many places difficult of application or adhesion, sillily throwing away, or wasting, a rather expensive substance.

III. It is a very common practice to cover the surface with powdered cantharides. *This should never be done*, but, instead, a few drops of olive or sweet oil put on the surface of the spread blister, the oil, by means of the finger, being rubbed into its surface and allowed to remain.

There are several objections to the practice of putting powdered cantharides on the surface of blisters. In the first place, a good one does not need it. In the second, a bad one wont be made good by its being there, as dry cantharides act very triflingly, or not at all, on the skin; and again, if the blister act it is troublesome to remove from the surface, thereby increasing the chances of stranguary by absorption, or forcing us to tear off the cuticle to obtain its removal.

The active property of Spanish fly is soluble in fixed oil. This, it may be said, is perfectly well known; possibly it is well known; but while the simple empl. canth. of the shops will act occasionally tolerably well in summer, in winter it is far otherwise; the vehicle is then almost solid, and my proposal is a means of obtaining the activity of the Spanish fly at all seasons, and independently of ordinary counteracting circumstances. Its activity by this means is not only rendered certain, but *more intense*, much less time being necessary to blister than usual, six to eight hours being quite enough, and even less will do where time is of importance.

The skin requires no preparation whatever—*stranguary never follows with me.* They are dressed afterwards in the ordinary way, with cotton. If the oil has been rubbed into the blister a few hours before being used, so much the better.

Circumstances, which I need not detail here, have forced me to have a blister on my own person this week. I used one as advised here. It rose beyond anything gentlemen, using the ordinary blister, in the ordinary way, are accustomed to see, and gave me little or no annoyance.—*London Lancet.*

